# Risk of Residual Neoplasia after a Local-Risk Resection of Colorectal Lesions by Endoscopic Submucosal Dissection: A Multinational Study

**DOI:** 10.3390/jcm12165356

**Published:** 2023-08-17

**Authors:** João Santos-Antunes, Mathieu Pioche, Felipe Ramos-Zabala, Paolo Cecinato, Francisco Gallego, Pedro Barreiro, André Mascarenhas, Sandro Sferrazza, Frieder Berr, Andrej Wagner, Arnaud Lemmers, Mariana Figueiredo Ferreira, Eduardo Albéniz, Hugo Uchima, Ricardo Küttner-Magalhães, Carlos Fernandes, Rui Morais, Sunil Gupta, Daniel Martinho-Dias, Isabel Faria-Ramos, Margarida Marques, Michael J. Bourke, Guilherme Macedo

**Affiliations:** 1 Gastroenterology Department, Faculty of Medicine, Centro Hospitalar Universitário S. João, 4200-319 Porto, Portugal; 2 Ipatimup/i3S (Instituto de Investigação e Inovação em Saúde da Universidade do Porto), 4200-135 Porto, Portugal; 3 Department of Hepatology and Gastroenterology, Edouard Herriot Hospital, 69003 Lyon, France; 4 Servicio de Gastroenterología, Departamento de Ciencias Médicas Clínicas, Hospital Universitario HM Montepríncipe, HM Hospitales, 28660 Madrid, Spain; 5 Gastroenterology and Digestive Endoscopy Unit, Azienda USL—IRCCS di Reggio Emilia, 42123 Reggio Emilia, Italy; 6 Gastroenterology Department, Hospital de Poniente, 04700 Almería, Spain; 7 Gastroenterology Department, Centro Hospitalar Lisboa Ocidental EPE, 1169-050 Lisbon, Portugal; 8 Lisbon Advanced Endoscopic Center, Hospital Lusíadas, 2724-002 Lisbon, Portugal; 9 Gastroenterology and Endoscopy Unit, Santa Chiara Hospital, 38123 Trento, Italy; 10 Department of Internal Medicine I, University Clinics Salzburg, Paracelsus Medical University, 5020 Salzburg, Austria; 11 Department of Gastroenterology, Hepatopancreatology and Digestive Oncology, CUB Erasme Hospital, Université Libre de Bruxelles (ULB), 1000 Brussels, Belgium; 12 Complejo Hospitalario de Navarra, Navarrabiomed Research Institute, Public University of Navarra, IdiSNA, 31006 Pamplona, Spain; 13 Servicio de Endoscopia Digestiva Centro Médico Teknon, 08022 Barcelona, Spain; 14 Servicio de Gastroenterología Hospital Universitario Germans Trias i Pujol, 08916 Barcelona, Spain; 15 Gastroenterology Department, Hospital Santo António, Centro Hospitalar Universitário do Porto, 4099-001 Porto, Portugal; 16 Gastroenterology Department, Centro Hospitalar de Vila Nova de Gaia/Espinho, 4400-129 Vila Nova de Gaia, Portugal; 17 Department of Gastroenterology and Hepatology, Westmead Hospital, Sydney 2145, Australia; 18 Department of Community Medicine, Information and Decision in Health, Faculty of Medicine, University of Porto, 4169-007 Porto, Portugal

**Keywords:** endoscopic submucosal dissection, colorectal lesions, lateral spreading tumor, residual lesion, piecemeal resection, local-risk resection

## Abstract

Endoscopic submucosal dissection (ESD) in colorectal lesions is demanding, and a significant rate of non-curative procedures is expected. We aimed to assess the rate of residual lesion after a piecemeal ESD resection, or after an en bloc resection but with positive horizontal margins (local-risk resection—LocRR), for colorectal benign neoplasia. A retrospective multicenter analysis of consecutive colorectal ESDs was performed. Patients with LocRR ESDs for the treatment of benign colorectal lesions with at least one follow-up endoscopy were included. A cohort of en bloc resected lesions, with negative margins, was used as the control. A total of 2255 colorectal ESDs were reviewed; 352 of the ESDs were “non-curative”. Among them, 209 were LocRR: 133 high-grade dysplasia and 76 low-grade dysplasia. Ten cases were excluded due to missing data. A total of 146 consecutive curative resections were retrieved for comparison. Compared to the “curative group”, LocRRs were observed in lengthier procedures, with larger lesions, and in non-granular LSTs. Recurrence was higher in the LocRR group (16/199, 8% vs. 1/146, 0.7%; *p* = 0.002). However, statistical significance was lost when considering only en bloc resections with positive horizontal margins (*p* = 0.068). In conclusion, a higher rate of residual lesion was found after a piecemeal ESD resection, but not after an en bloc resection with positive horizontal margins.

## 1. Introduction

Colorectal cancer is one of the most prevalent malignancies worldwide. In order to decrease the burden of colorectal cancer, efforts should be made to screen the population for early detection of the cancer and, preferably, to detect premalignant lesions. It is expected, due to the adenoma–adenocarcinoma sequence that is responsible for the majority of colorectal cancers, that malignancy incidence should decrease upon the resection of precursor lesions. There are two main endoscopic techniques for the resection of early gastrointestinal cancers and premalignant lesions. Endoscopic submucosal dissection (ESD) is now one of the mainstay endoscopic treatments for premalignant lesions of the digestive tract. This technique allows en bloc resection regardless of the lesion size and morphology, which is crucial for an accurate pathological evaluation and for a lower recurrence rate, as compared to a piecemeal resection by endoscopic mucosal resection (EMR). The selection of colorectal lesions for ESD is based on their size, morphology, and location and by a careful evaluation of the target lesions concerning their microvasculature and surface pattern, using classification systems such as Kudo classification, NICE, or JNET. Benign lesions or malignant lesions confined to superficial submucosa are usually candidates for endoscopic resection. Overall, very good clinical outcomes have been described using ESD in colorectal lesions [1,2,3]. However, the majority may still be managed by EMR, namely, those that are benign or with a limited suspicious area, which can be removed en bloc, with the remaining lesion being deliberately resected in several pieces [4]. ESD may be particularly important in lesions with higher risk of malignancy, in order to provide a cure from an oncological point of view [5,6,7,8]. Nevertheless, ESD is a complex procedure, it demands high endoscopic skills, and it has a long learning curve [9]. Therefore, a significant number of ESDs will present certain criteria that would classify them as non-curative resections.

Upon the diagnosis of a non-curative resection, clinicians and patients need to decide whether further therapeutic approaches should be applied, or if an endoscopic follow-up strategy would be enough, by a careful consideration of the risks and benefits of both strategies. The European Society of Gastrointestinal Endoscopy (ESGE) recently renamed “curative resections” as “very low risk resections” (VLRRs) or “low risk resections” (LRRs). Non-curative ESD (NC-ESD) are “local risk resections” (LocRR) or “high risk resections” (HRR) [10]. For colorectal lesions, VLRRs are benign lesions, en bloc resected, with free margins. LRRs are malignant (T1) with superficial submucosal invasion (SM1) and without high-risk features such as positive vertical margins, poor differentiation, or lymphovascular invasion, the presence of which would classify them as HRR. LocRRs are those piecemeal resected (benign component only) or those with dysplasia in the horizontal margins (HM+). For LocRR procedures, endoscopic follow-up is sufficient, while in HRR, complementary treatment, such as surgery or, in some cases, chemotherapy and/or radiotherapy, is usually warranted due to the risk of lymph node metastasis (LNM). 

The best strategy after an ESD defined as LocRR is not clear, since the guidelines mostly reflect an extrapolation of data from piecemeal or incomplete resection by EMR. However, positive horizontal margins after a complete endoscopic ESD may have a distinctive clinical significance due to the inherent differences in both techniques, namely, the ability to clearly identify the lateral margin of the lesion during the mucosal incision [11,12]. 

The aim of this study was to evaluate all the consecutive NC-ESDs performed in reference centers in the Western setting, assessing the rate of residual lesion during the endoscopic follow-up in benign lesions, and making comparisons to a control group of curative lesions, in order to better guide patients after LocRR.

## 2. Materials and Methods

### 2.1. Patient Selection

A retrospective, multicentric, multinational analysis of prospective registries of all the patients submitted to colorectal ESD between November 2009 and June 2021 was performed. Investigators with experience of more than 100 ESDs at the time of data collection were invited to participate with data from non-curative resections of benign colorectal lesions (piecemeal resected or en bloc resected with a positive horizontal margin). Fifteen centers from Portugal, Spain, France, Belgium, Italy, Austria, and Australia participated and contributed with these LocRR cases. 

The general indication for colorectal ESD was the presence of a colorectal neoplastic lesion without endoscopic features of deeply invasive (>SM1) adenocarcinoma [13,14]. For the purpose of this study, only patients with non-curative ESD (LocRR) performed for colorectal benign lesions (low-grade dysplasia (LGD) or high-grade dysplasia (HGD)) that had at least one follow-up endoscopy were selected for further analysis (case group). 

In parallel, consecutive ESDs performed in the coordinating center, Centro Hospitalar S. João, Porto, Portugal, were analyzed in order to retrieve all the curative resections of benign colorectal lesions (i.e., en bloc resected lesions with free horizontal and vertical margins)—VLRRs—for comparison (control group).

Patients gave their written informed consent before the procedure, and the Ethics Board of the coordinating center approved the study (255/2020), with clinicaltrials.gov identifier NCT04484311.

### 2.2. Definitions and Outcomes

Resection was considered en bloc whenever the target lesion was removed in a single piece, or it was considered piecemeal if the lesion was recovered in more than one fragment. Piecemeal ESD was considered whenever the lesion was removed in several pieces using only the ESD technique and devices. A knife-assisted resection (KAR)/hybrid technique was defined if a snare was used to complete the resection, after partial resection by the ESD technique. If pathological evaluation showed free margins in an en bloc resected specimen, we called it R0 resection; horizontal margins were only evaluated in en bloc resected specimens. For the purpose of statistical analysis, resections classified as HMx or VMx (due to artifacts that precluded a definite margin evaluation) were considered HM1 and VM1, respectively.

Colorectal curative resections (VLRRs) were those that were R0, with low- or high-grade dysplasia. All the other resections that were included (benign lesions with HM+ or piecemeal resection) were considered LocRRs [15]. 

Due to the retrospective design of the study, the histological report could not be standardized and centrally analyzed. However, international standards were used and applied by expert pathologists in each center. 

The major outcomes were the rate of residual dysplasia in the ESD scar, detected in follow-up endoscopies (and confirmed by biopsies and pathological analysis), after LocRR. Additionally, a comparison of the follow-up results between VLRRs and LocRRs was performed.

### 2.3. Statistical Analysis

An electronic database was created and filled by the investigators of the different centers. Absolute (n) and relative frequencies (%) were used to describe categorical variables. Continuous variables were described using means and standard deviations or medians and interquartile ranges (IQR), according to the normality of the distribution. Continuous variables were compared using Student’s *t* test or the Mann–Whitney test, while the chi-square test or Fisher’s exact test was used for categorical variables, as appropriate. Normality was verified using the Kolmogorov–Smirnov test to ensure correct test selection. Statistical analysis was performed using Statistical Package for the Social Sciences (SPSS) v.25.

## 3. Results

### 3.1. Patient Description 

A total of 2255 consecutive colorectal ESDs were reviewed. From them, 352 were considered “non-curative” ESDs, performed in either benign or malignant epithelial colorectal lesions that had at least one follow-up endoscopy. For the purpose of this study, 209 were LocRR and considered for evaluation: 133 HGD and 76 LGD. Of these, 10 were excluded due to missing data; therefore, a total of 199 benign colorectal lesions with LocRR were further included in the analysis.

**Figure 1:**
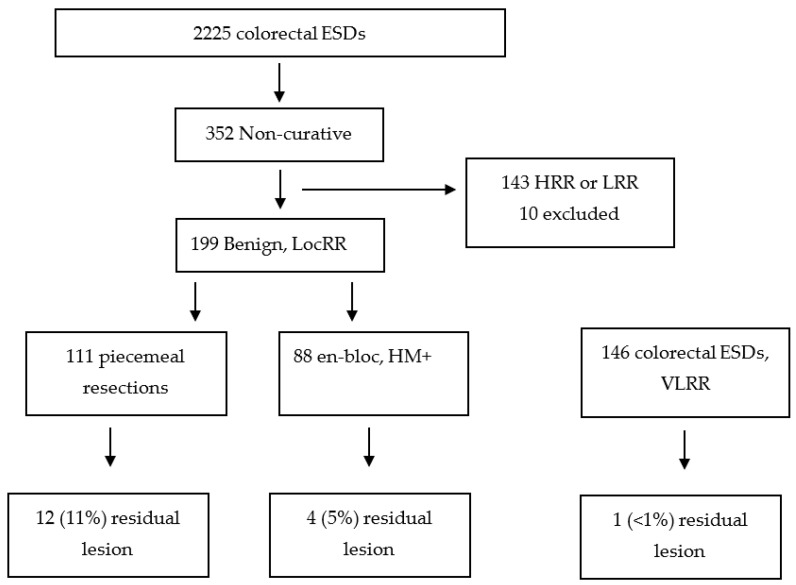
Flowchart of the patients included in the study. HRR—high-risk resection; LRR—low-risk resection; VLRR—very-low-risk resection; LocRR—local-risk resection; ESD—endoscopic submucosal dissection; HM—horizontal margin.

The prospective registry of the consecutive ESDs performed in the coordinating center was also analyzed, and a total of 146 curative resections (VLRRs), collected in the same time period, were used as a control group for comparison (Figure 1).

Considering all the analyzed patients (n = 345; 199 LocRRs and 146 VLRRs), 200 (58%) were male. The median lesion size was 40 mm (IQR 30–55 mm). In all, 141 (41%) lesions were located in the colon, and 204 (59%) were located in the rectum. As expected, most of the lesions were lateral spreading tumors (LSTs) of the granular, mixed-type morphology.

Procedural time, size, and other factors predicting a more complex procedure, such as the presence of the non-lifting sign and colonic location (vs. rectal location), were higher in the non-curative group. An analysis comparing baseline data between VLRR and LocRR can be found in Table 1.

### 3.2. Presence of Residual Lesions

Recurrence was higher in the LocRR group than in the VLRR group (16/199, 8% vs. 1/146, 0.7%; *p* = 0.002). However, statistical significance was lost when considering en bloc resections only (*p* = 0.068), contrary to what was observed in piecemeal-resected lesions (Table 2).

Among the 17 residual lesions, 15 were identified in the first follow-up, at 3–12 months. In one case, a residual lesion was verified at the second follow-up colonoscopy at 6 months, after an apparently negative colonoscopy at 3 months. In another case, a residual lesion was observed at the second follow-up after 30 months, after a negative colonoscopy at 6 months after the ESD.

## 4. Discussion

The NC-ESD project was first created to address the risk factors for the presence of residual disease after an NC-ESD, in either malignant or benign lesions, across the gastrointestinal tract. In this study, we focused on the non-curative procedure (LocRR) for the resection of benign colorectal lesions. In fact, this is the largest Western study concerning non-curative (LocRR) ESDs (n = 199) performed for benign colorectal lesions. We showed that piecemeal resection, but not positive horizontal margins, is associated with a higher rate of residual lesions in follow-up endoscopies. This result could have implications in the way we interpret pathological reports showing a positive horizontal margin in an endoscopically complete resection.

It is now widely accepted that ESD is very valuable for the treatment of colorectal neoplasia, and good outcomes have been described in Eastern and Western Countries [11,12,13]. ESD allows high rates of complete resection and a lower recurrence [14,15]. However, it is technically challenging, and a high rate of NC-ESDs is therefore expected. 

We analyzed a large multinational case series of NC-ESD procedures with a median endoscopic follow-up time of almost 2 years. According to a large metanalysis, 98% of residual lesions in the scar will be detected in the first year [16]. Hence, the endoscopic follow-up time in our study was sufficient for the detection of wall residual disease. 

Previous reports have shown that HM+ is the main cause of non-curative resection after an ESD for colorectal lesions [2,17], but the real clinical relevance for the patient is unclear. In fact, in the largest study to date on benign lesions with complete endoscopic resection but with positive horizontal margins (n = 96), recurrence was similar to that with HM0 [18], as we found in this study. 

ESD is a technique that allows the clear visualization of the lateral margin during the mucosal incision. Therefore, the presence of adenoma in the lateral margin in the ESD specimen should be unlikely. One explanation for the presence of a high rate of HM+ could be related to the post-ESD handling of the specimen. Since the margins of the lesions are easily identifiable, the endoscopist could perform the mucosal incision closer to the neoplastic tissue as compared to other organs. The small amount of normal tissue around the lesion may lead to an injury of the lateral margin by the pins when fixing it in the cork or rubber plate, causing the pathologist to falsely diagnose a positive horizontal margin. This may explain the low rate of recurrence and, therefore, the absence of clinical relevance of HM+ after a complete endoscopic resection by ESD. Widening the margins would lower the reported HM+ rates, but with doubtful clinical benefit, since outcomes between HM+ and HM0 are similar.

Data on colorectal ESD piecemeal-resected lesions are scarce, but this procedure seems to be a risk factor for recurrence in large lesions [19]. Besides this, it is well known that piecemeal resection by EMR is a risk factor for residual disease or recurrence [20,21,22]. However, piecemeal resection during an ESD procedure may be slightly different. It is known that coagulation of the borders of the eschar after a piecemeal EMR and the circumferential marking around the lesions before resection lower the risk of recurrence [23,24,25]. Therefore, we can assume that the presence of adenomatous tissue in the lateral margin could be the main reason for recurrent disease in a piecemeal EMR. However, the direct visualization of the mucosal incision during ESD should prevent the presence of the adenoma in the lateral margin, which could explain the very low rate of recurrence in en bloc resections, even with HM+ in the pathology report. It is possible that in some piecemeal ESDs, the use of salvage hybrid techniques to complete the resection with a snare was performed before the precise circumferential mucosal incision with the knife; this could explain the higher rates of recurrence, approximating those of piecemeal EMR. This should be further explored in prospective studies.

A previous meta-analysis showed a recurrence rate of 4% after an ESD, without considering the lateral margin status or whether the lesion was en bloc or piecemeal resected [26]. We found a recurrence rate below 1% in VLRR and of nearly 4% in lesions with positive horizontal margins, but a much higher rate in piecemeal ESD (almost 11%). Our findings, together with those of previous studies [18], challenge the ESD recommendation of performing a colonoscopy at 3–6 months after a resection with a positive horizontal margin; this surveillance may be only necessary for lesions resected in more than one fragment.

As expected, LocRRs were obtained in lengthier procedures, with larger lesions, with a much larger proportion of non-granular LSTs, with lesions that were already submitted to previous treatments and with the non-lifting sign, and with procedures that needed complementary techniques. Most of these are known risk factors for difficult and non-curative colorectal ESDs [6,27,28,29], so this baseline difference was expected in this retrospective analysis. 

Our study has some limitations. Its major strength is the very large number of Western centers that participated, allowing us to gather the largest Western case series on local-risk resections after a colorectal ESD. The main limitation is its retrospective nature, relying on prospective collected data, which could limit the interpretation of some data (for example, the distinction between a planned or salvage hybrid ESD). However, all the participating centers had a prospective ESD registry, which minimizes the risk of selection and information biases associated with observational and retrospective studies. Nevertheless, we must be aware that the fact that the VLRR lesion cases came from only one center may have resulted in selection bias, and the generalization of the results to other Western institutions is not straightforward since all the lesions in this study came from high-volume centers. Prospective multinational studies are now warranted to confirm these results and, eventually, to create predictive scores including not only non-curative criteria (such as piecemeal resection or positive margins) but also size and other morphological characteristics of the target lesion, as well as technical aspects of the ESD procedure, in order to better predict recurrence or residual disease and, consequently, to apply more accurate follow-up and therapeutic strategies.

## 5. Conclusions

In conclusion, we found a higher rate of residual lesions after piecemeal resection ESDs, but not after en bloc resection with a “positive horizontal margin”. Considering the absence of clinical consequences in our results, in agreement with those previously published, we think that there is enough evidence not to recommend a widening of the lateral margin in a colorectal ESD in order to have a higher rate of negative margins—which could lead to a higher rate of adverse events—nor to recommend stricter surveillance after en bloc resection with “positive horizontal margins”, provided that the endoscopist has the clear notion of a complete and radical resection during the ESD.

## Figures and Tables

**Table 1:** Comparison of VLRR and LocRR procedures.

	N	VLRR (n = 146)	LocRR (n = 199)	*p*
Male gender	200 (58%)	82 (56%)	64 (59%)	0.560
Female gender	145 (42%)	64 (44%)	81 (41%)	
Age (median, IQR)	-	66, 57–73	69, 61–75	**0.015**
ESD time (median, IQR)	-	90, 60–120	150, 120–190	**<0.001**
Lesion size (median, IQR)	-	40, 30–50	45, 32–60	**0.011**
LST classification				**<0.001**
. LSTGH	37 (15%)	13 (17%)	24 (15%)	
. LSTGNM	151 (63%)	59 (79%)	92 (55%)	
. NGFE	43 (18%)	3 (4%)	40 (24%)	
. NGPD	10 (4%)	0	10 (6%)	
Location				**<0.001**
. Colon	141 (41%)	29 (20%)	112 (56%)	
. Rectum	204 (59%)	117 (80%)	87 (44%)	
Previous attempt				**<0.001**
. Yes	60 (18%)	2 (1%)	58 (32%)	
. No	269 (82%)	144 (99%)	125 (68%)	
Non-lifting sign				**<0.001**
. Yes	65 (20%)	3 (2%)	62 (34%)	
. No	264 (80%)	143 (98%)	120 (66%)	
Complementary techniques				**<0.001**
. None	228 (71%)	117 (94%)	111 (57%)	
. KAR/hybrid	73 (23%)	0	73 (37%)	
. Clip-line	10 (3%)	4 (3%)	6 (3%)	
. Pocket	5 (2%)	0	5 (3%)	
. Underwater	5 (2%)	4 (3%)	1 (1%)	
Histology				0.866
. HGD	218 (63%)	93 (64%)	125 (63%)	
. LGD	127 (37%)	53 (36%)	74 (37%)	

VLRR—very-low-risk resection; LocRR—local-risk resection; ESD—endoscopic submucosal dissection; LSTGH—lateral spreading tumor, granular homogeneous type; LSTGMN—lateral spreading tumor, granular mixed-nodular type; LSTNGFE—lateral spreading tumor, non-granular flat-elevated type; LSTNGPD—lateral spreading tumor, non-granular pseudo-depressed type; KAR—knife-assisted resection; HGD—high-grade dysplasia; LGD—low-grade dysplasia.

**Table 2:** Comparison of the rate of residual disease between VLRR and LocRR procedures.

All Samples				
	Total n = 345	VLRR n = 146	LocRR n = 199	*p*
Follow-up time, months (median, IQR)		20 (14–40)	21 (8–24)	**0.011**
Residual lesion				**0.002**
. Yes	17	1 (0.7%)	16 (8.0%)	
. No	328	145 (99.3%)	183 (92.0%)	
**VLRR vs. piecemeal-resected lesions**				
	Total n = 257	VLRR n = 146	LocRR-p n = 111	*p*
Residual lesion				**<0.001**
. Yes	13	1 (0.7%)	12 (10.8%)	
. No	244	145 (99.3%)	99 (89.2%)	
**VLRR vs. en bloc resected lesions (with positive horizontal margin)**				
	Total n = 234	VLRR n = 146	LocRR-HM+ n = 88	*p*
Residual lesion				0.068
. Yes	5	1 (0.7%)	4 (4.5%)	
. No	229	145 (99.3%)	84 (95.5%)	

VLRR—very-low-risk resection; LocRR-p—local-risk resection (piecemeal resection); LocRR-HM+—local risk resection, positive horizontal margins.

## Data Availability

Data available on request from the corresponding author.
